# Driving key partner engagement by integrating community-engaged principles into a stakeholder analysis: A qualitative study

**DOI:** 10.1017/cts.2024.665

**Published:** 2024-11-27

**Authors:** Casey Allen, Emily Frankel, Shinobu Watanabe-Galloway, Heidi Keeler, Dave Palm, Brooke Fitzpatrick, Paul Estabrooks, Keyonna M. King

**Affiliations:** 1 University of Nebraska Medical Center, College of Medicine, Department of Neurological Sciences, Omaha, NE, USA; 2 University of Nebraska Medical Center, College of Public Health, Department of Epidemiology, Omaha, NE, USA; 3 University of Nebraska Medical Center, Office of Community Engagement, Omaha, NE, USA; 4 University of Nebraska Medical Center, College of Public Health, Department of Health Services Research & Administration, Omaha, NE, USA; 5 University of Utah, Department of Health and Kinesiology, Salt Lake City, UT, USA; 6 University of Nebraska Medical Center, College of Public Health, Department of Health Promotion, Omaha, NE, USA

**Keywords:** Stakeholder engagement, stakeholder analysis, community engagement, community-engaged Research, value proposition, qualitative methods, key partners, sustainable partnerships

## Abstract

**Introduction:**

The stakeholder analysis approach has historically been top-down rather than collaborative with key partners. However, this approach poses challenges for key partner engagement and community-engaged research, which aims to incorporate key partners throughout the project. This study, conducted by the Community Engagement Network at a Midwest Academic Medical Center, seeks to examine the value of community-engaged research for diverse key partners to increase collaboration, strengthen partnerships, and enhance impact, ultimately driving key partner engagement.

**Methods:**

The study involved semi-structured interviews with 38 key partners from diverse groups, including community members, community organizations, Practice-Based Research Network members, researchers, research administration, university administration, and potential funders. The interview guide, informed by an extensive literature review, assessed perceived value, barriers, and improvement strategies for community-engaged research, supplemented by value proposition statements.

**Results:**

The analysis revealed three main themes: 1) Fostering Community Buy-In: Authentic representation and inclusive partnerships were essential for trust and commitment; 2) Enhancing Communication and Dissemination: Effective communication strategies were vital for maintaining engagement and sharing research outcomes; and 3) Building Capacity and Ensuring Sustainability: Continuous learning and long-term investments were crucial for sustaining community-engaged research efforts.

**Discussion:**

This study underscores the value of incorporating key partners into stakeholder analyses to enhance collaboration, strengthen partnerships, and improve the impact of community-engaged research. The findings offer valuable insight for institutional transformation and implementation of effective stakeholder analyses and engagement tools, ultimately enhancing the effectiveness of research strategies and initiatives.

## Introduction

Engaging key partners as equal members of a research team stimulates collaboration and enhances the overall impact of research. Key partner engagement, defined as “the meaningful involvement of patients, caregivers, and other healthcare stakeholders through the entire research process,” is both a moral imperative and a strategic approach that fosters reciprocal relationships, co-learning, transparency, and trust [1,2]. This engagement strengthens partnerships and broadens the scope of research to be more inclusive and effective. We define key partners as “…a person or group of persons, who are influenced by or able to influence the project” [3]. By adopting this terminology instead of “stakeholders,” we aim to contribute to the decolonization of research and move beyond language that contradicts the principles of community engagement [4]. Our team will be utilizing the term “key partners” to refer to our “stakeholders” and “stakeholder groups.” We will continue to use the term “stakeholder” when referring to referenced works, tools, or frameworks where the term is used (e.g., stakeholder analysis, stakeholder theory, etc.). Effective key partner engagement enhances the quality and sustainability of community-engaged research projects, supports strong partnerships, expands knowledge, and ultimately improves health programs and policies for thriving communities [5–7].

**Figure 1.**
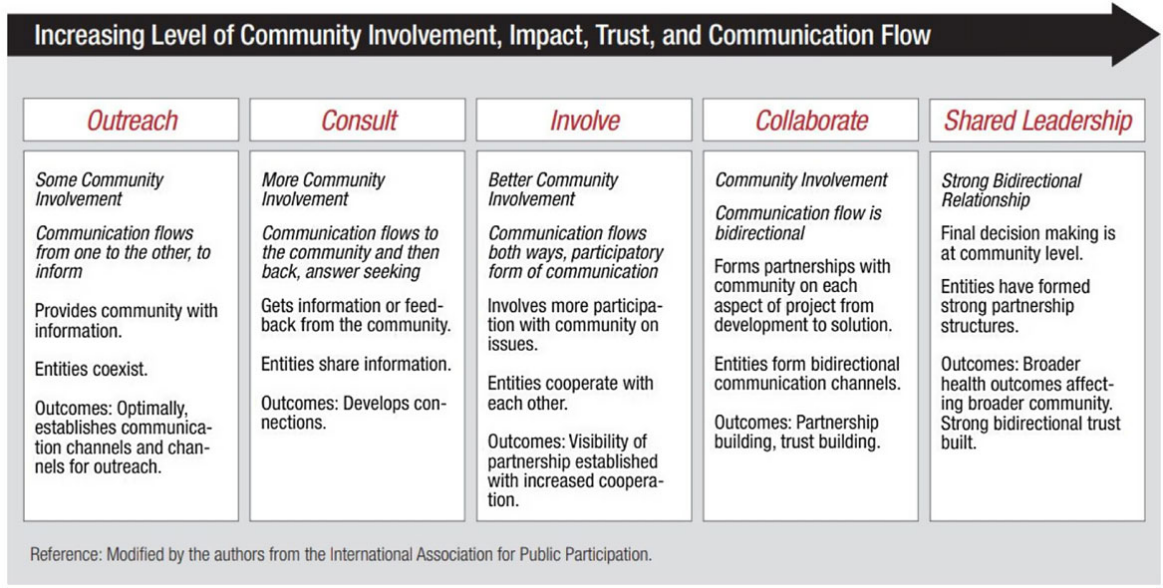
Community engagement continuum.

Although heterogeneous in its application, key partner engagement must abide by the principles of community engagement and strive for the highest level of engagement to yield the greatest impact [8,9]. The nine principles of community engagement described by the Clinical and Translational Science Award Consortium Community Engagement Key Functions Committee Task Force on the Principles of Community Engagement are outlined in Table 1. These principles are incorporated in Figure 1 to demonstrate levels of engagement [9].

**Table 1.** Nine principles of community engagement

Be clear about the purposes or goals of the engagement effort and the populations and/or communities you want to engage.
Become knowledgeable about the community’s culture, economic conditions, social networks, political and power structures, norms and values, demographic trends, history, and experience with efforts by outside groups to engage it in various programs. Learn about the community’s perceptions of those initiating the engagement activities.
Go to the community, establish relationships, build trust, work with formal and informal leadership, and seek commitment from community organizations and leaders to create processes for mobilizing the community.
Remember and accept that collective self-determination is the responsibility and right of all people in a community. No external entity should assume it can bestow on a community the power to act in its own self-interest.
Partnering with the community is necessary to create change and improve health.
All aspects of community engagement must recognize and respect the diversity of the community. Awareness of the various cultures of a community and other factors affecting diversity must be paramount in planning, designing, and implementing approaches to engaging a community.
Community engagement can only be sustained by identifying and mobilizing community assets and strengths and by developing the community’s capacity and resources to make decisions and take action.
Organizations that wish to engage a community as well as individuals seeking to effect change must be prepared to release control of actions or interventions to the community and be flexible enough to meet its changing needs.
Community collaboration requires long-term commitment by the engaging organization and its partners.

A stakeholder analysis is a commonly used process for increasing engagement that identifies key partners and provides a better understanding of their positionality surrounding a given project, policy, or topic. This process can be used to enhance key partner engagement and community-engaged research through a deeper understanding of key partners’ perceptions, values, and needs surrounding a project [10,11].

Stakeholder analyses have been used since Freeman introduced the subject in 1984 [3]. Freeman’s stakeholder theory expanded the understanding of key partners to include anyone affected by a given company and its workings, suggesting that success is wider than simply capitalistic profit [3]. By identifying and understanding the perspectives and interests of different key partner groups, organizations can enhance decision-making, foster collaboration, and mitigate potential conflicts.

Stakeholder analyses can be broken down into four general steps: 1) brainstorming for key partner groups, 2) identifying categories of key partners and understanding why they are important, 3) prioritizing key partners, and 4) understanding your key partners [12]. When identifying key partners, the PESTLE model can be a helpful tool to utilize through the brainstorming process [12]. The PESTLE model recognizes Political, Economic, Sociological, Technological, Legal, and Environmental influences of power [13]. After a list of key partners has been identified, it’s important to consider how each person or group relates to your project via their specific interests, be it positive or negative [12]. The third step of stakeholder analysis proceeds to prioritize key partners in relation to “their importance and influence” [12]. Key partners with higher levels of influence or interest can help to push project progress along faster when included earlier in the project timeline. The final step of the stakeholder analysis process collates the information collected across the preceding steps to create a more well-rounded understanding of key partner interests, impacts, and risks pertaining to a given project [12].

The stakeholder analysis approach has historically been top-down rather than collaborative with key partners, due to the goal of gaining insight into the interests, power, and positionality of key partners surrounding a goal pertaining to a project or policy [14]. However, this approach poses challenges for key partner engagement and community-engaged research, which aims to incorporate partners throughout the project. Limitations of the traditional approach include marginalizing key partners’ voices and relying on assumptions rather than real-time community input [14,15].

The literature on community engagement and stakeholder analysis outside the context of organized projects is limited. However, evidence suggests that re-centering the research to focus on key partners can enhance the sustainability of community engagement and ongoing relationships [16,17]. By integrating community engagement principles into stakeholder analyses, a more participatory, holistic process can emerge, providing a well-rounded understanding of key partners and strategies for improving engagement and integration [9]. Through a discourse analysis, this paper aims to qualitatively examine the value of community-engaged research for diverse partners to increase collaboration, strengthen partnerships, and enhance impact, ultimately driving key partner engagement.

## Materials and methods

### Interview guide

To accomplish our stated aim, the research team, comprised of the Community Engagement Network (CEN) at a Midwest Academic Medical Center, reviewed existing literature on key partner, patient, and community engagement in research to understand the value of diverse participation in research as it relates to contexts, processes, and outcomes. Based on the findings of the literature, we generated 3–6 value propositions for our identified key partner groups, described in “Interviewee Selection,” around principles of knowledge, capacity, trust, decision-making, social progress, personal development, and collaboration [18–21]. These value propositions incorporate the principles of community engagement, described in Table 1. A value proposition is “a clear, simple statement of the benefits, both tangible and intangible, that the evidence-based program/intervention will provide to a particular key partner, along with a recognition of the approximate financial, time, implementation, and other costs associated with the benefits” [22]. The value propositions can be found in Table 2.

**Table 2.** Value proposition statements

Key Partner Category	Value Proposition Statements
Community Members	Community members value community-engaged research when: There is potential to improve your community. You can contribute to designing, implementing, and completing projects to benefit where you live or work. It gives you opportunities for capacity building or personal development. It aligns with your personal interests. Research focuses on fairness, justice, empowerment, broad participation, applicability, and self-determination.
Community Organizations	Community organizations value community-engaged research when: Research aligns with and advances your mission. You can partner and be fully involved in research to identify important questions that have the potential to help the community achieve its goals. You have opportunities to lead or co-lead the research. There are resources available to support communities in getting the research done. It improves your organizational capacity.
Practice-Based Research Network (PBRN) Members	Practice-Based Research Networks value community-engaged research when: There are resources to improve clinic operations. The research answers clinically meaningful questions. The research improves provider and patient experiences, processes of care, and health outcomes. You can partner to co-produce and lead clinically relevant research projects. The research is bi-directionally beneficial—it makes the research easier and the outcomes from the research make the services more impactful and useful.
Researchers	Researchers value services that support community-engaged research when: They can help recruitment and retention goals. They make your research results more relevant and impactful. They help with dissemination of findings and results. They help secure extramural research funding. They give opportunities for new collaboration. They help build a good reputation for you or your institution in the community.
Research Administration	Research administration value services that support community-engaged research when: It increases extramural funding. It makes it easier to recruit and retain hard to reach participants. It creates a trusting and sustainable partnership to continue research. It improves overall research metrics for the institution.
University Administration	University Administration value services that support community-engaged research when: It meets the needs of community groups. It improves the reputation of the institution in the community. It creates a trusting and sustainable partnership with local community organizations. It informs and develops a community engagement structure.
Potential Funders	Potential funders value services that support community-engaged research when: It meets the needs of community groups. Community voices are heard and acted on. The outcomes and milestones are important to the community and the funding agency objectives.

In addition to the value propositions, the interview guide assessed three content areas: 1) perceived value and/or benefit of participating in community-engaged research, 2) perceived barriers to participating in community-engaged research, and 3) ways to improve the impact of community-engaged research for key partners. General demographic information, including key partners’ occupation, organization of employment, and how they currently participate in community-engaged research was also gathered. In total, the interview guide consisted of 14 open-ended questions, followed by 4–7 value proposition statements, resulting in interviews lasting 20–45 minutes.

### Interviewee selection

To mirror the first step of a traditional stakeholder analysis, we generated a purposive sample of key partner groups that play an integral role in community-engaged research. The key partner groups identified for desired perspectives included community members, community organizations, Practice-Based Research Network members, researchers, research administration, university administration, and potential funders. Members of the CEN then generated a list of potential interviewees that represented each key partner group, based on preexisting partnerships. Once the key partner list was created, five names were randomly selected from each key partner group, using the website random.org, to ensure each key partner had an equal chance to be selected and to prevent selection bias from the CEN members. We believe this approach would generate a more robust and representative sample in each key partner group. A total of 40 individuals were invited to participate in the key partner interviews, 38 interviews were completed, and 2 individuals did not respond to the invitation to participate. Table 3 displays the distribution of interviewees across key partner categories.

**Table 3.** Distribution of interviewees across key partner categories

Key Partner Category	Number of Interviewees
Community Members	5
Community Organizations	6
Practice-Based Research Network (PBRN) Members	5
Researchers	6
Research Administration	6
University Administration	5
Potential Funders	5
**Total**	**38**

### Conducting interviews

Interviewers were affiliated with the Community Engagement and Outreach Core of the Great Plains IDeA Clinical and Translational Research Network (*n* = 4), Community Outreach and Engagement Core of the Fred and Pamela Buffett Cancer Center (*n* = 1), and the university’s Office of Community Engagement (*n* = 2). Informal interviews were conducted virtually between November 2021 and February 2022, using the Zoom web conferencing platform. The use of Zoom met HIPAA Compliance standards. Interviews were conducted using the interview protocol described above and recorded with the interviewees’ permission. Data saturation was established when no novel insights were uncovered in the interview. All interviews were transcribed into Research Electronic Data Capture (REDCap) upon completion. The interviewers brought diverse community engagement perspectives and experiences to the research team through their educational backgrounds, previous experience, and intersectional identities, leading to unique and valuable bias throughout the interview process. Personal characteristics of interviewers can be found in Table 4.

**Table 4.** Research team and reflexivity

Interviewer	Credentials	Gender	CEN Affiliation
1	PhD, MSN, MBA, RN	Female	UNMC OCE
2	MPH	Female	CTR CEO
3	PhD	Female	BCC COE
4	PhD	Male	CTR CEO
5	DrPH, MA	Female	CTR CEO
6	MPH	Female	UNMC OCE
7	PhD, MS	Male	CTR CEO

Community Engagement Network (CEN) Affiliation: UNMC OCE-University of Nebraska Medical Center Office of Community Engagement; CTR CEO-Great Plains IDeA-CTR Community Engagement and Outreach; BCC COE-Buffett Cancer Center Community Outreach and Engagement.

Modified from the Consolidation Criteria for Reporting Qualitative Studies (COREQ)[23].

The Office of Regulatory Affairs at the University of Nebraska Medical Center determined that this project did not constitute human subjects research as defined by 45CFR46.102 and was therefore not subject to federal regulations.

### Data analysis

Members of the CEN, comprising the data analysis team, developed an etic codebook using an inductive approach prior to completing our coding analysis of the interview notes. We utilized this approach to allow for themes to emerge. We summarized the themes that emerged from our coding tree in Table 5. Two research team members coded the interview notes independently. Their coding results had a high level of agreement, indicating consistency across the coding process. When there was a coding disagreement, the team discussed it until general consensus was reached. After the coding was completed, the research team pulled illustrative quotes that aligned with the themes. The CEN team reviewed the results for accuracy and completeness.

**Table 5.** Interview themes and subthemes

Themes and subthemes	Illustrative Quotation
*Fostering community buy-in*	
Authentic representation of community perspectives and identities	“would value CER that reflects the diversity of the community where that community [engaged research] is being done”
Inclusivity & collaborative partnerships	“There is a very large push from the leadership. We need buy-in from different levels. Institutions provide support especially junior faculty or people who want to transfer into that space is needed.” “Inclusivity: respectful approach to research that involves people in the community. Shows that we care about various viewpoints. Shows respect for inclusion that we care about the partnership with the community.” “It is important to communicate new research opportunities and obtain buy-in from the community.” “Community members want to participate AND receive the feedback.” “Including the community as often as possible and early.” “The relationships to do those things is extremely important because without them you don’t have buy-in.” “Finding new partners to expand research opportunities and finding new venues for creating awareness.”
*Enhancing communication & dissemination*	
Continuity through past research updates	“Citizen Advisory Board to update about the past studies that the board approved – to see where things are at. – e.g., Citizen Scientists testing water – what happened? Additional outcomes or research?”
Sustaining ongoing engagement	“Finding opportunities to connect regularly to ensure all involved in this work knows what others are doing. So many individual efforts makes it confusing for community members.”
Community-centered dissemination	“Having information disseminated; administrators get removed and understanding and hearing community needs is critically important – dissemination of health information to community.” “Give a platform to share the results of research. (Refugee) communities need to know.” “Some way to share data seamlessly without much pain from the community side and our side.”
Balancing research & action	“Goes back health disparities – the only way to find solutions is to do this work in the community. We need to do this research to find solutions to the problems.” “Are we working on the right problems [that are] meaningful to the community - which speaks to measuring. What are we measuring and can we show it’s getting better. It [is] not just about a paper or grant but we show that an intervention increased X or Y in a community and how to scale it up. Strategies we devise and how sustainable they are in the community.”
*Building capacity & ensuring sustainability*	
Continuous learning for key partners	“Training and best practices in community engagement. Value community engagement that can also benefit students” “Educate people on the benefits of sharing the good experiences & elevate it!” “More training in community-engaged research.” “More diverse workforce will be helpful”
Long-term investments	“More grassroots work working directly with the community, not just the leaders.” “We need to continue to evolve the work, not just stop it and leave the community hanging.” “Capacity. More time to do things and learn to understand; have more time to do engagement.”

CER-Community-Engaged Research.

The analysis aimed to contribute to the ongoing discussion on key partner engagement by demonstrating the value of qualitative data in enriching the stakeholder analysis process. The findings are projected to inform both theoretical frameworks and practical strategies for key partner engagement, ultimately enhancing the effectiveness of organizational strategies and initiatives reliant on stakeholder analyses or other engagement tools.

## Results

A total of 38 interviews were conducted. The analysis of these interviews yielded three overarching themes: 1) fostering community buy-in, 2) enhancing communication and dissemination strategies, and 3) building capacity and ensuring sustainability. The themes and subthemes are available alongside their illustrative quotations in Table 5.

### Theme 1: Fostering community buy-in

Within community engagement initiatives, fostering community buy-in emerges as a pivotal theme, essential for nurturing trust and genuine commitment from key partners across projects. This not only revolves around authentically representing diverse community perspectives and identities but also cultivating inclusive and collaborative partnerships. These subthemes underscore the imperativeness of creating spaces where all voices are heard, respected, and actively engaged, ultimately contributing to the co-creation of impactful solutions that resonate with the community’s needs, goals, and aspirations.

#### Authentic representation of community perspectives and identities

Partners expressed that authentic representation is fundamental to the success of any community engagement effort. It underscores the significance of genuinely valuing and incorporating diverse perspectives and identities within communities. In discussions with partners, the importance of this aspect was consistently highlighted:

*“…would value [community engaged research] that reflects the diversity of the community where that community [engaged research] is being done.”*

This concept transcends mere acknowledgment; it involves intentional incorporation of a comprehensive process that emphasizes meaningful community engagement in decision-making processes. According to scholarly literature, authentic representation is best defined as a multifaceted approach that seeks to involve community members in shaping policies, programs, and initiatives that directly impact them [2].

Recognizing and respecting the richness of community perspectives plays a pivotal role in enhancing innovation, building and maintaining trust, fostering a sense of belonging, and ultimately securing genuine buy-in for research initiatives. By actively involving community members in the decision-making process, organizations can demonstrate their commitment to inclusivity and ensure that their efforts are truly reflective of the community’s needs and aspirations. One participant further emphasized this point:

*“The relationships to do [community engaged research] is extremely important because without them you don’t have buy-in.”*

#### Inclusivity and collaborative partnerships

Partners believed these types of partnerships further reinforced the importance of dedicated relationship-building prior to research activities. Incorporating collaboration throughout the research timeline fosters a sense of shared ownership and responsibility. Successful research initiatives are characterized by a collective, participatory approach that actively involves community members in decision-making processes. This was conveyed by one participant who shared that relationships are crucial in community-engaged research, as they facilitate buy-in from community members who not only want to participate but also receive feedback on their involvement:

*“Community members want to participate AND receive the feedback.”*

### Theme 2: Enhancing communication & dissemination

Partners expressed the significance of enhancing communication and dissemination efforts within the context of community-engaged research. Effective communication not only facilitates engagement but also ensures that research findings reach diverse audiences, fostering greater trust, understanding, and impact within the community. This theme unfolds through four significant subthemes: 1) continuity through past research updates, 2) sustaining ongoing engagement, 3) community-centered dissemination, and 4) balancing research and action.

#### Continuity through past research updates

Ensuring continuity through past research updates is essential for fostering community engagement and trust. Partners expressed a desire to be informed about previous research outcomes, emphasizing the value of building on past efforts by maintaining a continuous flow of updates. Consistent and accessible information regarding the progression of research initiatives not only contributes to building a sense of transparency and knowledge sharing but also acknowledges and incorporates historical context. Past research informs and enriches current strategies, providing a foundation for ongoing collaboration and improvement.

*“…update about the past studies that the [Citizen Advisory] board approved – to see where things are at.”*

#### Sustaining ongoing engagement

Sustaining ongoing engagement requires dynamic communication that goes beyond one-time interactions. Partners emphasized the importance of effective, iterative communication to ensure that all key partners involved in the work are informed and connected. They highlighted the need for regular opportunities to connect and share updates, particularly in contexts where multiple individual efforts may create confusion for community members. One participant stated:

*“Finding opportunities to connect regularly to ensure all involved in this work know what others are doing. So many individual efforts make it confusing for community members.”*

#### Community-centered dissemination

Community-centered dissemination strategies are crucial for ensuring that research findings resonate with, and are accessible to, the community. This involves tailoring communication materials, formats, and channels to align with community values, language, and preferences. Effective dissemination efforts must incorporate community members to ensure relevance and accessibility, which provides a platform for sharing research results and facilitating meaningful dialogue as a couple of participants pointed out:

*“Give a platform to share results of research. …communities need to know.”*

*“…some way to share data seamlessly without much pain from the community side and our side.”*

#### Balancing research and action

Partners expressed that this sub-theme is paramount, as research is not only about gathering and conveying knowledge but also mobilizing towards collaborative and impactful actions. Partners emphasized the importance of using strategies that clearly convey research findings to inspire tangible, community-driven actions. They stressed the need to focus on addressing health disparities and finding solutions within the community, thus showcasing interventions that lead to measurable improvements and advocating for effective sustainable strategies that can be scaled up. One participant stated:

*“…Goes back [to] health disparities – the only way to find solutions is to do this work in the community. We need to do this research to find solutions to the problems.”*

Another participant further described why it is important to involve community in research and the positive impact it can produce on sustainability:

*“Are we working on the right problems [that are] meaningful to the community-which speaks to measuring. What are we measuring and can we show it’s getting better? It [is] not just about a paper or grant but we show that an intervention increased X or Y in a community and how to scale it up. Strategies we devise and how sustainable they are in the community.”*

### Theme 3: Building capacity and ensuring sustainability

Participants expressed the importance of building capacity to conduct community-engaged research not just for researchers, but also for community. In addition, many of the participants discussed the importance of universities and other academic collaborators committing to a long-term investment in sustaining community partnerships beyond the scope of a grant period. As a result, we identified two key subthemes: 1) continuous learning for key partners and 2) long-term investments.

#### Continuous learning for key partners

Participants emphasized how essential continuous learning is for fostering effective community engagement and collaboration. Some participants emphasized the importance of ongoing education and skill development across research teams, therefore leveling the playing field among community and academic partners. Spreading awareness of community-engaged research and fostering research environments conducive to continuous learning are vital. This may involve integrating training programs, workshops, and knowledge-sharing dissemination platforms to empower all key partners and enhance their capacity for meaningful participation. Key partners have expressed the need for training in best practices in community engagement, recognizing the value it brings to both the community and students involved in research endeavors:

*“Training best practices in community engagement. Value community engagement that can also benefit students.”*

*“More training in community-engaged research.”*

#### Long-term investments

Participants expressed that long-term investments are crucial for sustaining authentic community partnerships and ensuring the sustainability of community-engaged research efforts. However, there were several challenges described by participants related to funding and the sustainability of relationships at the conclusion of a project, which can disrupt community partnerships and key partner engagement if not adequately addressed. Recognizing the time commitment involved in community-engaged research, there is a call for long-term investment strategies that diversify the community members engaged across various levels. A forward-looking approach that extends beyond the grant or research timeline is necessary to cultivate stability and capacity-building within the community. This approach circles back to the notion of fostering a sense of ownership and empowerment among community leaders, key partners, and participants. Key partners emphasized the importance of grassroots work, which provides direct engagement with the community, and the need for ongoing evolution and adaptation of research efforts to meet community needs and sustain meaningful engagement:

*“More grassroots work working directly with the community, not just the leaders.”*

*“We need to continue to evolve the work, not just stop it and leave the community hanging.”*

*“Capacity. More time to do things and learn to understand; have more time to do engagement.”*

## Discussion

Our study qualitatively examined the value of community-engaged research for diverse partners to increase collaboration, strengthen partnerships, and enhance impact, ultimately driving key partner engagement. We explored key partners’ perceptions and reactions to stakeholder analyses by specifically assessing their perceived value, barriers, and improvement strategies for community-engaged research with value proposition statements. We analyzed the data by reading the interview notes several times looking for illustrative statements of the perceptions that key partners held when asked about proposition statements related to traditional stakeholder analyses and community-engaged research. Our analysis revealed three main themes that, we believe, will enhance key partner engagement by increasing collaboration, partnerships, and impact.

To our knowledge, there is no peer-reviewed literature describing the integration of community into a traditional stakeholder analysis framework. Therefore, the significance of this study lies in its methodological approach, which emphasizes the importance of incorporating community engagement principles into the stakeholder analysis process to increase key partner engagement in subsequent community-engaged research projects. By directly engaging key partners and creating space for them to express their perceived value of community-engaged research based on their experience, this research seeks to provide insights that are not only contextually grounded but also reflective of the complex realities that key partners navigate across the community engagement continuum.

Our findings highlight the critical importance of fostering community buy-in for the success of community-engaged research. Authentic representation of community perspectives emerged as a fundamental requirement. Participants emphasized that community engagement efforts must genuinely value and incorporate diverse perspectives and identities within communities. This aligns with existing literature which underscores that genuine community representation enhances innovation, trust, and a sense of belonging, characteristics that are commonly missing from traditional research [24]. Our study extends this understanding by demonstrating that inclusive and collaborative partnerships are not merely beneficial, but essential for gaining and maintaining community commitment. These insights suggest that institutions must prioritize building and nurturing authentic relationships with community members throughout a research timeline to ensure effective engagement.

Effective communication and dissemination strategies were identified as pivotal for sustaining ongoing engagement and ensuring that research findings resonate with and are accessible to the community. Key partners expressed a strong desire for continuity through regular updates on past research, which builds transparency and trust. This finding supports the idea that community members are not passive recipients of information but active partners who seek continuous engagement [20]. Furthermore, community-centered dissemination strategies, which tailor communication to align with community values and preferences, were deemed crucial. These strategies not only facilitate better understanding and impact but also empower communities to engage in meaningful dialog about research findings. This approach highlights the importance of balancing research and actionable outcomes, ensuring that research initiatives lead to tangible, community-driven actions. It also provides insights about how to meaningfully strategize the engagement of key partners when conducting a stakeholder analysis.

Building capacity and ensuring sustainability were also highlighted as essential for the long-term success of community-engaged research. Continuous learning opportunities for both researchers and community members were seen as vital for fostering effective engagement and collaboration. Participants stressed the need for ongoing education and skill development, which supports the notion that capacity-building efforts are crucial for leveling the playing field between community and academic partners [18]. Additionally, long-term investments in community partnerships were deemed necessary to sustain engagement beyond the scope of individual projects or grant periods. This finding underscores the importance of a forward-looking approach that extends beyond immediate research goals to cultivate stable and enduring community relationships that are necessary to consider in the stakeholder analysis process.

The insights gained from this study have significant implications for both practice and future research. Practitioners should prioritize community engagement as part of the stakeholder analysis process because it can result in authentic representation and inclusive partnerships in their engagement strategies. Effective communication and dissemination efforts must be community-centered, ensuring that research findings are accessible and actionable. Capacity-building initiatives should focus on continuous learning for all key partners involved, fostering a collaborative research environment.

This study’s use of purposive sampling limited the captured perspectives of interviewees to those who already had a relationship with a member of the research team. This approach likely excluded individuals who are not typically involved in research or who are not in an active partnership or study. Additionally, the development of the interview guide relied heavily on existing literature available on stakeholder analyses which typically rely on an etic approach. This limits the emic insights we were able to glean from our data analysis. Future research should explore community engagement in stakeholder analyses to establish increased long-term impact of sustained community engagement on research outcomes and community well-being. Additionally, there is a need to develop standardized methods for participatory stakeholder analyses that can be integrated with traditional approaches to provide a comprehensive understanding of key partner dynamics through both an etic and emic lens.

This study underscores the value of incorporating qualitative insights into stakeholder analyses to enhance collaboration, strengthen partnerships, and improve the impact of community-engaged research. By engaging key partners directly and allowing them to express their views, this research provides contextually grounded insights that are crucial for the success of key partner engagement strategies. The findings offer valuable insight for institutional transformation and implementation of effective stakeholder analyses and engagement tools, ultimately enhancing the effectiveness of research strategies and initiatives. Best practices in community-engaged research suggest the need to decolonize the language surrounding engagement by incorporating strategies that are inclusive of key partners’ backgrounds, experiences, and assets [4]. We strongly encourage our peers in the field to adopt the term “key partners” to further this effort. This work demonstrates valuable strategies for integrating the principles of community engagement in qualitative methodologies.
